# The Prognostic Impact of the Aryl Hydrocarbon Receptor (AhR) in Primary Breast Cancer Depends on the Lymph Node Status

**DOI:** 10.3390/ijms20051016

**Published:** 2019-02-26

**Authors:** Udo Jeschke, Xi Zhang, Christina Kuhn, Stéphan Jalaguier, Jacques Colinge, Kristina Pfender, Doris Mayr, Nina Ditsch, Nadia Harbeck, Sven Mahner, Sophie Sixou, Vincent Cavaillès

**Affiliations:** 1 LMU Munich, University Hospital, Department of Obstetrics and Gynecology, 81377 Munich, Germany; zhang.xi@sz.tsinghua.edu.cn (X.Z.); Christina.kuhn@med.uni-muenchen.de (C.K.); kpfender@web.de (K.P.); nina.ditsch@med.uni-muenchen.de (N.D.); nadia.harbeck@med.uni-muenchen.de (N.H.); sven.mahner@med.uni-muenchen.de (S.M.); sophie.sixou@inserm.fr (S.S.); 2 Tsinghua Berkeley Shenzhen Institute, Tsinghua University, Shenzhen 518055, China; 3 IRCM, Institut de Recherche en Cancérologie de Montpellier, INSERM U1194, 34298 Montpellier, France; stephan.jalaguier@inserm.fr (S.J.); jacques.colinge@inserm.fr (J.C.); vincent.cavailles@inserm.fr (V.C.); 4 Université de Montpellier, 34000 Montpellier, France; 5 Institut régional du Cancer de Montpellier, 34298 Montpellier, France; 6 LMU Munich, Department of Pathology, 80337 Munich, Germany; doris.mayr@med.uni-muenchen.de; 7 Faculté des Sciences Pharmaceutiques, Université Paul Sabatier Toulouse III, 31062 Toulouse CEDEX 09, France; 8 Cholesterol Metabolism and Therapeutic Innovations, Cancer Research Center of Toulouse (CRCT), UMR 1037, Université de Toulouse, CNRS, Inserm, UPS, 31037 Toulouse, France

**Keywords:** AhR, breast cancer, lymph node, luminal-like, RIP140, prognostic marker

## Abstract

Increasing evidence implicates the aryl hydrocarbon receptor (AhR) as a possible regulator of mammary carcinogenesis. This study aims to clarify its prognostic impact in breast cancer (BC). Meta-analyses performed at the mRNA level demonstrated that the predictive value of AhR expression in BC depends on the lymph node (LN) status. AhR expression and sub-cellular location were then analyzed by immunohistochemistry in 302 primary BC samples. AhR was expressed in almost 90% of cases with a predominant nuclear location. Nuclear and cytoplasmic AhR levels were significantly correlated and associated with the expression of RIP140 (receptor-interacting protein of 140 kDa), an AhR transcriptional coregulator and target gene. Interestingly, total and nuclear AhR levels were only significantly correlated with short overall survival in node-negative patients. In this sub-group, total and nuclear AhR expression had an even stronger prognostic impact in patients with low RIP140-expressing tumors. Very interestingly, the total AhR prognostic value was also significant in luminal-like BCs and was an independent prognostic marker for LN-negative patients. Altogether, this study suggests that AhR is a marker of poor prognosis for patients with LN-negative luminal-like BCs, which warrants further evaluation.

## 1. Introduction

Breast cancer (BC) is a global public health issue and the most commonly diagnosed neoplasm in the world [1]. The expression of estrogen receptor α (ER), progesterone receptor (PR), and human epidermal growth factor receptor 2 (HER2) is widely accepted as prognostic and predictive markers and thus considered suitable to guide treatment decisions (endocrine- or anti-HER2-targeted therapies) [2]. However, BC is an extremely complex and heterogeneous disease with different clinical behavior and disease evolution. Therefore, the development of additional specific markers associated with BC malignancy and disease prognosis is clearly needed.

The aryl hydrocarbon receptor (AhR) belongs to the basic helix-loop-helix Per-Arnt-Sim homology domain family [3]. AhR is a transcription factor which is activated by ligands (such as dioxin [4]) and translocates from the cytoplasm to the nucleus. It dimerizes with ARNT (AhR nuclear transporter), leading to the regulation of target genes implicated in several diseases, including cancer [5]. The transcriptional coregulator RIP140 (Receptor Interacting Protein of 140 kDa) interacts with AhR and acts as a coactivator of its ligand-induced activity [6]. Interestingly, RIP140 also appears to be an AhR target gene in BC cells, since its expression is induced by dioxin [7].

AhR is involved in a variety of cellular processes (such as cell cycle regulation, epithelial barrier function, cell motility and migration, immune function) required to maintain cell homeostasis [3,8]. Dysregulation of these processes leads to tumor initiation, promotion, and progression. AhR and AhR-regulated genes have been implicated as potential actors in BC progression [9]. AhR expression was reported to be higher in tumor cells than in normal breast tissue and to be associated with histological type and p53 status [10]. In a recent study, the expression of AhR was observed in the cytoplasm and nucleus of tumor cells and in their microenvironment, with high levels being correlated with the high expression of several genes related to inflammation and invasion [11]. AhR is expressed in both hormone receptor-positive and -negative BCs, including triple-negative BCs (TNBC) [12]. AhR expression is not only correlated with type and stage of invasive BCs [13], but also with prognosis, including overall survival (OS) and distant metastasis-free survival [12].

To decipher the specific role of AhR expression in BC, we first set up a meta-analysis of AhR mRNA expression using various cohorts of BC patients stratified according to the lymph node (LN) status. We then retrospectively analyzed AhR immunoreactivity and its clinical significance, in a cohort of 302 tumors from primary BC patients, again stratified according to LN status. For this, we combined the quantification of nuclear and cytoplasmic AhR levels and performed correlation analyses with clinicopathological features, with the expression of an AhR coregulator/target gene, and finally with patient survival.

## 2. Results

### 2.1. Analysis of AhR Expression in BC at the mRNA Level

**Figure 1:**
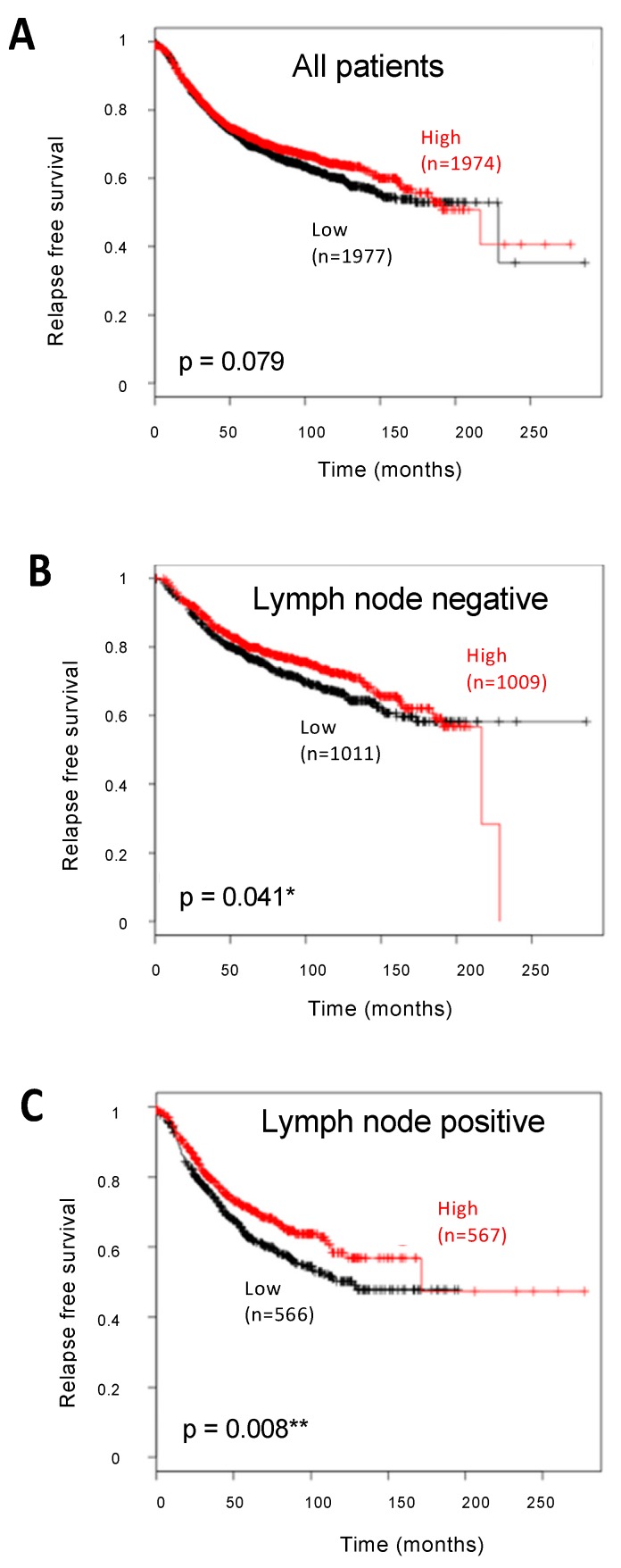
Survival of patients with breast cancer (BC) according to aryl hydrocarbon receptor (AhR) mRNA expression and lymph node status. Kaplan–Meier analysis of the correlation between AhR mRNA expression with relapse-free survival in all (**A**), lymph node-negative (**B**), or lymph node-positive (**C**) BC patients. A median cut-off value was used. The number of cases in each arm is indicated in each panel, for low (in black) or high (in red) AhR mRNA expression. Correlations are statistically significant for *p* < 0.05 (*) and for *p* < 0.01 (**).

As a first attempt to correlate AhR expression with prognosis in BC, we used the Kaplan–Meier Plotter online database to perform Kaplan–Meier analyses on several BC transcriptomic datasets. As shown in Figure 1, the meta-analysis showed that the levels of AhR mRNA (assessed using the 202820_at probeset) were not significantly associated with prognosis (*n =* 3951 patients). Interestingly, the correlation was highly significant in LN-positive patients (*n =* 1133, *p =* 0.008), and stronger than in LN-negative patients (*n =* 2020, *p =* 0.041). Using the BreastMark algorithm (Supplementary Figure S1), AhR mRNA levels were significantly associated with OS only in LN-positive patients (*n =* 473, *p =* 0.002). These results suggest that AhR expression may influence patient survival according to the LN status.

### 2.2. Immunodetection of AhR in BC

We then analyzed AhR expression at the protein level by immunohistochemistry using a cohort of 302 BC tissues from 297 primary BC patients, with clinical data shown in Table 1. Among the 297 patients, five were bilateral primary BC, so we dealt with the tumors as individual cases (*n =* 302). Follow-up ranged from 10–12 years and the median age was 57.5 years old. Several analyses have been performed previously in our group on this cohort, with results dealing with N-Cadherin (NCAD) and CD133 [14], nuclear receptors [15,16], and their transcriptional coregulators RIP140 and LCoR [17].

Staining for AhR was obtained for 302 primary BC tissues and observed in the nucleus and/or cytoplasm of tumor cells. The evaluation in each subcellular location was performed with a standardized immunoreactive score (IRS) ranging from 0 to 12 [18]. Total AhR expression was calculated as the sum of nuclear and cytoplasmic IRS.

**Figure 2:**
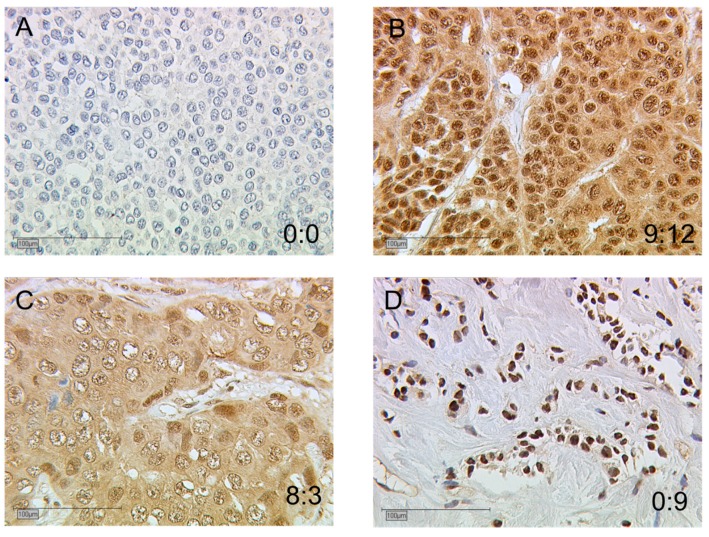
Immunohistological staining of AhR in primary BC samples. AhR expression is illustrated in four tumors (**A**–**D**) with null or low nuclear expression (**A**,**C**) and high nuclear expression (**B**,**D**). The cytoplasmic:nuclear immunoreactive score (IRS) ratios are indicated for each sample. Scale bars: 100 μm.

In Figure 2, four examples of AhR staining are displayed. For each photograph, the cytoplasmic: nuclear ratio is given with low and/or high IRS values. These samples showed that AhR nuclear and cytoplasmic stainings were not systematically correlated in all tumors. Indeed, besides low (A) or high (B) nuclear and cytoplasmic IRS, tumors exhibiting low nuclear with high cytoplasmic IRS (C) or high nuclear with low cytoplasmic IRS (D) were also observed.

The mean IRS values of total, nuclear, and cytoplasmic AhR are presented in Table 2 in the total cohort (*n =* 302). Based on the data obtained at the mRNA level, results are also presented in the two sub-groups corresponding to LN-negative or -positive patients (*n =* 162 and 124, respectively, with 16 being unknown). The data demonstrated that nuclear AhR expression was significantly stronger (*p* < 0.05) than cytoplasmic expression in all sub-groups (4.15 ± 2.92 vs. 2.51 ± 2.67 in the whole cohort), whereas AhR expression was not significantly influenced by the LN status.

For the comparison of tumors expressing low or high AhR IRS values, we used optimized cut-off values determined by receiver operating characteristic curve (ROC-curve) analysis based on OS. The optimized cut-off values were IRS ≥ 2 for total AhR and IRS ≥ 1 for either nuclear or cytoplasmic AhR. As indicated in Table 2, high AhR immunoreactivity was present in a large proportion of BC tissues (91.1% of tumor cells in the whole cohort, and similar percentages in the LN-negative or -positive sub-groups). We observed more tumors with nuclear AhR (n *=* 275, 91.1%) than with cytoplasmic AhR staining (*n =* 177, 58.6%) in the whole cohort, and similarly in the LN-negative and -positive sub-groups (*p* < 0.05).

Correlation coefficients were calculated for AhR expression in the nuclear and cytoplasmic cell compartments (Table 2). Using a Spearman Rho test, we demonstrated that nuclear and cytoplasmic AhR stainings were statistically strongly correlated in the whole cohort and in the LN-negative and -positive sub-groups. Total AhR staining was obviously also strongly correlated with either nuclear or cytoplasmic staining (data not shown).

### 2.3. Analysis of the Correlation between AhR Expression and Clinicopathological Characteristics and RIP140 Expression

We then analyzed the correlations between AhR expression and various relevant clinicopathological parameters, including age at time of diagnosis; histologic type; tumor size; grade; local recurrence; distant metastasis; expression of ER, PR, and HER2; and TNBC status. By analyzing the whole cohort as well as the LN-positive and -negative sub-groups, we found no significant correlation between total AhR expression and the different parameters (Table 3). It should be mentioned that no correlation was observed with the expression of other nuclear receptors, including VDR, RXR, and THRα1 (data not shown).

On the contrary, a significant correlation appeared with the expression of the AhR target gene and the transcriptional coregulator RIP140 (*p =* 10^−7^, 1.1 10^−3^ and 5.6 10^−4^ in the whole cohort, and in the LN-negative and -positive sub-groups, respectively), in the whole cohort and in the two LN sub-groups (Table 3). When analyzing nuclear and cytoplasmic AhR expression, very similar correlations with RIP140 were obtained (Supplementary Table S1)—high correlations persisting with nuclear and cytoplasmic AhR in the whole cohort and in the two sub-groups. Of note, RIP140 was better correlated with nuclear (*p* < 0.01) than cytoplasmic AhR (*p* < 0.05) in the LN-negative sub-group. Altogether, these data supported the previously described regulation of RIP140 gene expression by AhR [7].

*Correlation of Total and Nuclear AhR levels with Patient Survival*

**Figure 3:**
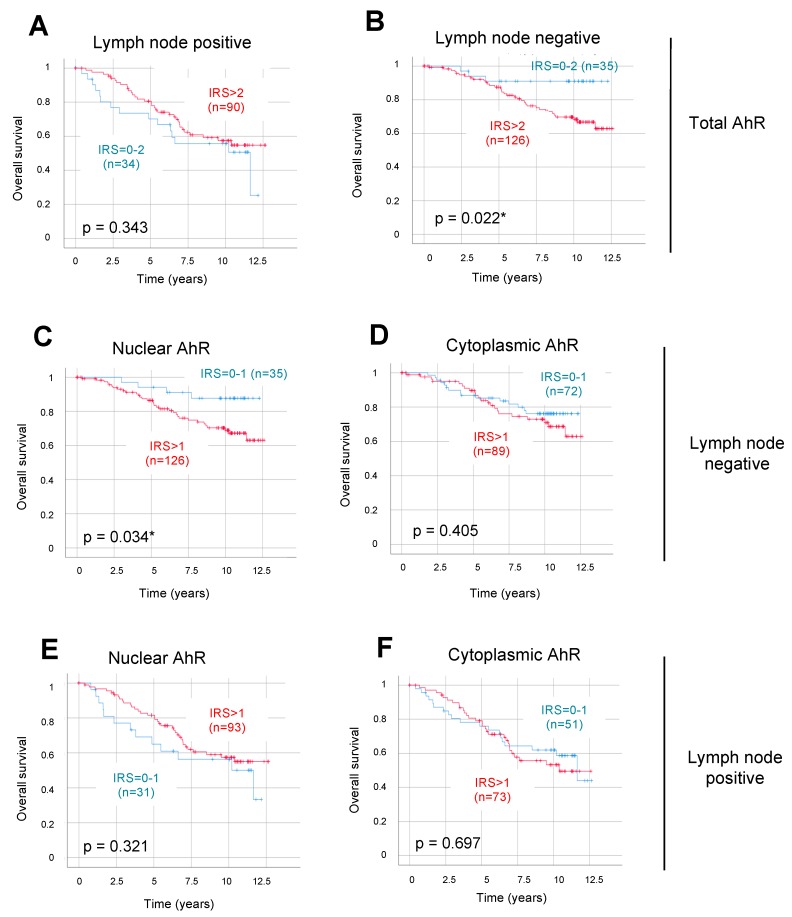
Overall patient survival according to total, nuclear, and cytoplasmic AhR expression in lymph node-negative versus lymph node-positive BC. Kaplan–Meier analysis of the correlation between OS and total AhR expression in lymph node-positive (**A**) or lymph node-negative (**B**) patients, and nuclear or cytoplasmic AhR in lymph node-negative (**C** and **D**, respectively) or lymph node-positive patients (**E** and **F**, respectively). The IRS cut-off values together with the number of cases in each arm are indicated in each panel, for low (in blue) or high (in red) AhR expression. Correlations were statistically significant for *p* < 0.05 (*).

Using Kaplan–Meier analysis, we then analyzed correlations between AhR protein expression and OS in the whole patient cohort and in the two BC sub-populations (i.e., LN-negative and -positive). To compare cohorts with low or high IRS values, we used the optimized cut-off values as defined above. Considering the whole cohort, no statistically significant correlations with OS were found with the expression of total, nuclear, or cytoplasmic AhR expression (data not shown). We then performed stratifications of the cohort, based on LN status. Total AhR expression was not correlated with OS in patients with LN-positive BC (*n =* 124, *p =* 0.343) (Figure 3A). Very interestingly, a significant inverse correlation with OS was observed in patients with LN-negative BC (*n =* 161, *p =* 0.022) (Figure 3B).

Focusing on the LN-negative sub-group of BC patients, we found that nuclear AhR expression had a strong and significant negative impact on OS (*p =* 0.034, Figure 3C). By contrast, cytoplasmic AhR was not correlated with OS in the sub-group of patients with LN-negative BC (Figure 3D). Moreover, neither nuclear nor cytoplasmic AhR had any correlation with OS in patients with LN-positive tumors (Figure 3E,F, respectively). Therefore, our results demonstrated that nuclear (and total) AhR was a negative prognostic factor for OS only in LN-negative patients.

*Low RIP140 Expression Improves the Correlation of AhR Levels with Survival*

**Figure 4:**
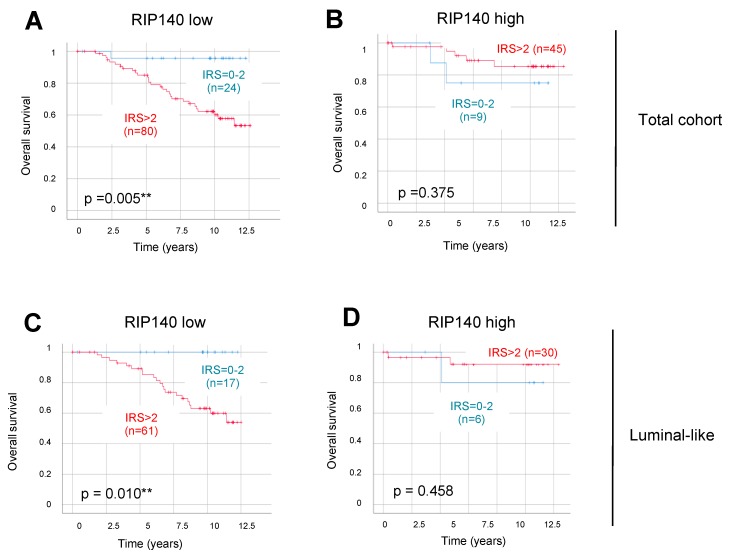
Overall patient survival according to total AhR expression in lymph node-negative BC, according to RIP140 expression. Kaplan–Meier analysis of the correlation between OS and total AhR expression in lymph node-negative patients, with either a low (**A**,**C**) or high (**B**,**D**) RIP140 expression. The analysis has been performed with all lymph node-negative patients (**A**,**B**) and with only the luminal-like lymph node-negative patients (**C**,**D**). The IRS cut-off value is above 4 for RIP140 and for AhR, and IRS cut-off values together with the number of cases in each arm are indicated in each panel, for low (in blue) or high (in red) AhR expression. Correlations are statistically significant for *p* < 0.01 (**).

In order to improve the prognostic impact of AhR, we then stratified the sub-group of LN-negative patients according to low and high RIP140 expression as a marker of AhR transcriptional activity. Using a cut-off IRS value of 4 for RIP140 expression, we observed that OS was shorter for patients with tumors, harboring high total AhR expression combined with low RIP140 levels (*p =* 0.005, Figure 4A). No significant correlation was observed in the high RIP140-expressing sub-group (*p =* 0.375, Figure 4B). As for the total AhR, the expression of nuclear AhR combined with low RIP140 levels was significantly correlated with a short OS (*p =* 0.005, Supplementary Figure S2A). Neither cytoplasmic AhR in the low RIP140-expressing sub-group (Supplementary Figure S2C), nor nuclear and cytoplasmic AhR in the high RIP140-expressing sub-group (Supplementary Figure S2B,D, respectively) had any correlation with OS. The same stratification in the LN-positive sub-groups did not reveal any negative correlation (data not shown). Altogether, these data suggested that the AhR^high^/RIP140^low^ signature might help to identify poor prognosis patients with short survival amongst node-negative BC patients, i.e., in patients who are commonly believed to have a rather good prognosis.

Our hypothesis was that this selection might also exist among patients with an even better initial prognosis. With that in mind, we decided to concentrate on luminal-like tumors by eliminating from the cohort those patients who had a more aggressive tumor biology, i.e., those with HER2-positive BC or TNBC. Very interestingly, the correlation of total AhR expression with short OS was still strong and significant in the sub-group of luminal-like tumors expressing low RIP140 (Figure 4C, *p =* 0.010). As for the total cohort, no significant correlation was observed in tumors with a high level of RIP140 (Figure 4D, *p =* 0.458). Of note, all 17 patients with AhR^low^/RIP140^low^ luminal-like tumors presented in the same Figure 4C survived at least 12.5 years after the first BC diagnosis. The same stratification in the LN-positive sub-groups did not reveal any significant correlation, although we noticed the opposite trend in most cases (Supplementary Figure S3A). Indeed, patients with high AhR expression exhibited an apparently, but not statistically, significant better OS than those with low AhR expression, especially in the low RIP140-expressing sub-group of luminal-like tumors (Supplementary Figure S3C, *p =* 0.056). In conclusion, these results suggest that the combination of total AhR levels with a low expression of RIP140 identifies sub-groups of BC patients with very different survival profiles. This prognostic impact of the AhR^high^/RIP140^low^ signature is applicable to patients with luminal-like tumors.

### 2.4. AhR Expression as an Independent Prognostic Parameter in LN-Negative Tumors

Finally, we performed multivariate analyses for the whole cohort and for patients with positive or negative LN status using the Cox regression model with total AhR expression and various clinicopathological features (age at time of diagnosis, tumor size, ER, and HER2 status). As shown in Table 4, data demonstrated that, in the whole cohort, only age, tumor size, ER, and HER2 were independent prognostic markers. Very interestingly, total AhR appeared as an independent prognosis marker only in the LN-negative sub-group of patients (*p =* 0.046), with a hazard ratio of 3.369, indicating a much higher risk of death for the patients with tumors expressing high levels of AhR.

## 3. Discussion

The purpose of this study was to define the prognostic impact of AhR in BC. We correlated its expression with several parameters, including clinicopathological features, RIP140 expression (as a marker of AhR transcriptional activity), and patient outcome.

The data we generated from our patient cohort demonstrated that the AhR gene exhibited substantial protein expression in the vast majority of tumors analyzed. This further supports the hypothesis of AhR activation and overexpression in BC compared to normal breast tissues [10,19,20]. In our study, AhR was predominantly expressed in the nuclei, with a strong correlation with cytoplasmic expression (Figure 2). AhR is a protein which exhibits a nucleocytoplasmic shuttling mediated by nuclear localization and export signals [21,22,23], with specific roles in each compartment related to its partner ARNT [24,25]. In non-small cell lung cancer cells, in the absence of agonist ligands, resting AhR expression is predominantly cytoplasmic and acts as an epithelial mesenchymal transition (EMT) suppressor via a non-genomic pathway [26]. In a study of 90 patients with invasive ductal breast carcinoma, AhR was expressed in the cytoplasm with higher expression than in normal duct epithelium [10,19]. Interestingly, in a rat model of mammary tumorigenesis, besides the modest cytoplasmic localization of AhR in normal myoepithelial and ductal cells, nuclear AhR was predominantly expressed in tumors, and associated with the upregulation of the *CYP1* target gene, suggesting constitutive AhR activation already at early stages of mammary tumorigenesis [20].

Concerning the correlation between AhR expression and BC patient survival, our data clearly indicated that this correlation depends on the LN status of the tumor. Indeed, although AhR protein levels were not related to OS in the whole cohort, its total and nuclear expressions were significantly associated with poor outcome in LN-negative disease (Figure 3B and 3C, respectively). More interestingly, we demonstrated that total AhR is an independent prognostic marker only in the sub-group of LN-negative patients (Table 4). It is known that adjuvant systemic therapy of primary BC patients may have an effect on the prognostic impact of a given marker [27]. We are therefore aware that the correlation we observed could be biased due to the effects of adjuvant systemic therapy, usually used for most LN-positive patients at the time of diagnosis. However, because of the diversity of the systemic treatments used, we could not consider this parameter in the present study.

Interestingly, in LN-positive patients, the opposite trend is observed, although not statistically significant, with a rather beneficial effect of AhR protein expression on patient OS (see, for instance, Supplementary Figure S3C). This trend was in accordance with the data observed at the mRNA level. Indeed, Figure 1C and Supplementary Figure S1B demonstrated a statistically significant beneficial effect of high AhR mRNA expression in the LN-positive patients. These data may suggest that the correlation of AhR with patient prognosis may depend on whether the metastatic process is engaged or not. In addition, this differential correlation of AhR with survival may be linked to its ligand-dependent or -independent activations. Investigations highlight various potential underlying mechanisms of AhR actions in BCs, including the regulation of EMT [28]. Interestingly, AhR has been reported to exert positive or negative effects on EMT [26]. In a previous study performed in the same cohort of patients, we demonstrated that NCAD, as an EMT marker, negatively correlated with the OS in the LN-negative sub-group of patients [14]. Consequently, we searched for a correlation between AhR expression and NCAD. In the two sub-groups of tumors, LN-positive or -negative, NCAD was strongly correlated with cytoplasmic AhR. Interestingly, in patients combining LN-positive and low NCAD expression, high nuclear AhR levels indicated significantly better OS (data not shown).

For patients with LN-negative BC, we demonstrated that the correlation of total and nuclear AhR with poor OS was stronger when expression of the RIP140 gene was low (Figure 4A and Supplemental Figure S2A, respectively). The same results were obtained with disease-free survival (DFS) analysis (data not shown). AhR was reported to be strongly associated with RIP140 acting as a coactivator of its transcriptional activity [6], which might explain the crosstalk between AhR and ERα [29]. Moreover, the RIP140 gene is an AhR target since RIP140 mRNA expression is induced by liganded AhR in BC cells [7]. In BC patients, we recently demonstrated that high total and cytoplasmic RIP140 protein levels were correlated with shorter DFS, whereas high nuclear RIP140 was correlated with longer OS [17]. It should be mentioned that no significant differences, in terms of correlation between RIP140 mRNA or protein expression and patient survival (OS or DFS), were observed between LN-negative and LN-positive tumors (data not shown). The present study demonstrates that, in the subgroup of LN-negative patients, RIP140 status displays a significant association with AhR expression (Table 3). We could hypothesize that low RIP140 expression might reveal non-functional AhR nuclear signaling and this subgroup of tumors might reflect a role of AhR in the nucleus, independent of its ligand-activated functions. Another possibility is that the AhR/RIP140 complex regulates different sets of genes in LN-negative and LN-positive tumors. Alternatively, the stratification of LN-negative tumors according to RIP140 levels might simply help in identifying the tumors with the best overall prognosis in which AhR levels appeared to be the most discriminating.

Our data clearly demonstrated that the AhR^high^/RIP140^low^ signature was significantly associated with poor prognosis for patients with luminal-like and LN-negative tumors in our cohort (Figure 4C). Interestingly, in this sub-group, no death was observed during 12.5 years among the 17 patients with AhR^low^/RIP140^low^-expressing tumors. Moreover, by focusing on early primary luminal-like BC (pT1 and pT2 only, tumor size inferior to 5 cm), the same significant correlation of total AhR expression and short OS was observed (*p =* 0.013, data not shown). Altogether, the AhR^high^/RIP140^low^ signature seems to stratify patients with luminal-like and node-negative BCs in two groups with very good or poor OS.

Currently, the decision to treat BC patients by chemotherapy is currently driven by taking into account various clinicopathologic factors, such as patient age, disease stage, tumor grade, ER, and PR status, as well as the overexpression or amplification of HER2 (Adjuvant!Online and PREDICT tools) [30]. For patients with luminal-like LN-negative BCs, several multiparameter genomic assays are available, including Oncotype DX, MammaPrint, Prosigna, or EndoPredict. Our data suggest that the AhR/RIP140 signature may also help in better stratifying this subgroup of patients. Of interest, neither AhR nor RIP140 belong to the different mRNA signatures that are currently available. This is not surprising considering the strong difference between AhR mRNA and protein regarding their respective correlation with OS.

## 4. Materials and Methods

### 4.1. Correlation of Gene Expression with Patient Survival

The Kaplan–Meier plotter (http://kmplot.com) allows meta-analysis-based biomarker assessment using a background database which is manually curated [31]. To analyze the prognostic value of a particular gene, the patient samples are split into two groups according to median expressions. The two patient cohorts are compared by a Kaplan–Meier survival plot, and the hazard ratio with 95% confidence intervals and a log rank *p*-value are calculated. The 202820_at probe set was used for AhR mRNA and relapse-free survival was taken as the endpoint (Figure 1).

BreastMark (http://glados.ucd.ie/BreastMark) is an algorithm allowing the identification of genes that are associated with disease progression in BC [32]. The search was performed at a low cut-off threshold with OS as the survival endpoint. Survival curves are based on Kaplan–Meier estimates and the log rank *p*-value is shown for difference in OS (Supplementary Figure S1).

### 4.2. Collective

We used a well-characterized collective of 302 paraffin-embedded breast tumor tissue samples, from 297 primary BC patients (five of them are bilateral BC). All patient data were fully anonymized and all diagnostic procedures had already been fully completed when samples were collected for the study. Authors were blinded from the clinical information during the experimental analysis. The study was approved by the Ethical Committee of the Ludwig Maximilians University of Munich, Germany (LMU, approval number 048–08 Date: 18. 03. 2008) and informed consent was obtained from all patients. Tissues were sampled between 2000 and 2002 from patients treated for BC at the Department of Obstetrics and Gynaecology of the LMU. Tumor stage at primary diagnosis was histologically evaluated using the UNICC tumor node metastasis (TNM) classification, which includes the size and extent of tumors (primary tumor size, or pT, classified as: pT1, pT2, pT3, pT4), involvement of regional LN (pN), and presence or absence of metastasis (M). The tumor grade was determined by an experienced pathologist (Dr D. Mayr) of the Department of Pathology of the LMU, according to a modification of Elston and Ellis grading proposed by Bloom and Richardson [33]. Additional data, such as ER and PR status, age, histological grade, metastases, local recurrence, progression, and survival, were retrieved from the Munich Cancer Registry. HER2 expression was analyzed with an automated staining system (Ventana; Roche, Mannheim, Germany), according to the manufacturer’s instructions. Cases were considered negative for 0 or 1+ stainings, and positive for 3+ stainings. For all cases with 2+ staining, amplification was confirmed by FISH (fluorescence in situ hybridization). Among the 302 tumors, 232 (78.8%) were from primary BC patients without metastatic evolution, and 59 (19.5%) were from patients who became metastatic during the follow-up.

### 4.3. Immunohistochemistry

Expression of ERα, PR, and HER2 was determined at diagnosis, in all BC samples of this cohort at the LMU Department of Pathology, Germany. ERα and PR expression were evaluated by immunohistochemistry, as described previously [33]. Samples showing nuclear staining in more than 10% of tumor cells were considered as hormone receptor-positive, in agreement with the guidelines at the time of the analysis (2000–2002). HER2 expression was analyzed with an automated staining system (Ventana; Roche, Mannheim, Germany), according to the manufacturer’s instructions. Data on VDR, RXR, and THRα1 expression in these BC samples were extracted from a previously published study [34], as well as for NCAD, CD133 [14], and RIP140 and LCoR [17]. For AhR analysis, samples were processed as previously described [14,17,35]. Paraffin-embedded tissue was used for the preparation of 3 μm sections. For the staining procedure, tissue sections were dewaxed in xylol (Carl Roth GmbH & Co. KG, Karlsruhe, Germany) for 15 min at room temperature. In order to reduce the endogenous peroxidase reaction, sections were immersed in a solution of 3 % hydrogen peroxide (VWR International S.A.S., Fontenay-sous-Bois, France) in methanol (Sigma-Aldrich, Steinheim, Germany) for 20 min. After rehydrating in decreasing concentrations of ethanol (100–0% in distilled water), slides were boiled for 5 min in a pressure cooker with sodium citrate buffer (pH 6) for epitope retrieval. Then, the sections were washed with distilled water and phosphate buffered saline (PBS), before blocking with Powerblock (Biogenex, San Ramon, CA, USA) in distilled water (1:10) for 3 min. Tissue sections were then incubated (16 h at 4 °C) with a polyclonal rabbit antibody against AhR (Abnova, Aachen, Germany) at a 1:200 dilution and washed again in PBS. After incubation with a corresponding biotinylated secondary anti-rabbit IgG antibody, and with the associated avidin-biotin-peroxidase-complex (both Vectastain Elite ABC Kit; Vector Laboratories, Burlingame, CA, USA), visualization was performed with substrate and chromogen 3, 3-diamino-benzidine (DAB; Dako, Glostrup, Denmark). Negative and positive controls were used to assess the specificity of the immunoreactions. Negative controls (colored in blue) were performed in BC tissue by replacement of the primary antibodies by species-specific (rabbit) isotype control antibodies (Dako, Glostrup, Denmark). Appropriate positive controls (placenta samples) were included in each experiment. Sections were counterstained with acidic hematoxylin and afterward dehydrated in increasing concentrations of ethanol (70–100%). The slides were immediately mounted with Eukitt (Merck, Darmstadt, Germany) before manual analysis with a Diaplan light microscope (Leitz, Wetzlar, Germany) with 2.5×, 10× or 40× magnifications. Pictures were obtained with a digital CCD camera system (JVC, Tokyo, Japan).

### 4.4. Data Analysis

For all proteins, status was classified by evaluation of the percentage of brown stained tumor cells and staining intensity, allowing the assessment of an IRS, as a product of multiplication between the positive cells proportion score (0–4) and staining intensity score (0–3) [18]. For the percentage score of stained tumor cells, we considered no cells stained (score *=* 0), <10 % stained (score *=* 1), 10–50 % stained (score = 2), 51–80 % stained (score = 3), and 81–100 % stained (score = 4). Staining intensity was evaluated as no (score = 0), weak (score = 1), moderate (score = 2), and strong (score = 3) stainings. In doubtful cases, slides were evaluated by two or three independent examiners with consent at the end. As previously described for LCoR and RIP140 [17], AhR cytoplasmic and nuclear stainings were evaluated in parallel, with the determination of both cytoplasmic IRS and nuclear IRS separately. The total IRS was calculated by the addition of cytoplasmic and nuclear IRS. For all other markers, staining and IRS were determined in the whole cells, without differentiation of nuclear and cytoplasmic staining.

### 4.5. Statistical and Survival Analysis

Statistical analyses were performed using SPSS 24 (IBMSPSS Statistics, IBM Corp., Armonk, NY, USA). For all analyses, *p* values below 0.05 (*), 0.01 (**), or 0.001 (***) were considered statistically significant. To present the mean immunoreactivity levels described by the IRS in Table 2, the groups were divided into low- vs. high-expressing AhR cases and according to the LN status. We then performed receiver operating characteristic curve (ROC) analyses to calculate the optimal cut-off values between low and high AhR expression, based upon the maximal differences of sensitivity and specificity. The threshold determined regarding OS was an IRS ≥ 1 for either nuclear or cytoplasmic AhR, and ≥ 2 for total AhR. These thresholds were used to determine the percentages of tumors expressing low or high AhR levels described in Table 2, besides the OS analysis detailed below.

Correlation analyses presented in Table 2 and Table 3 and Supplementary Table S4 were performed by calculating the Spearman’s-Rho correlation coefficient (*p* values of Spearman’s-Rho test presented).

Survival times were compared by Kaplan–Meier graphics and differences in OS were tested for significance by using the chi-square statistics of the log rank test (Figure 3 and Figure 4, and Supplementary Figures S2 and S3). Data were assumed to be statistically significant in the case of *p*-value < 0.05. Kaplan–Meier curves and estimates were then provided for each subgroup and each marker, as well as for the combined expression of two markers in order to compare the differences of survival times between LN-negative and -positive patients and between low and high AhR and other marker expression. The *p* value and the number of patients analyzed in each subgroup are given for each chart.

Multivariable analysis for outcome (OS) presented in Table 4 was performed using the Cox regression model, and included AhR expression and relevant clinicopathological characteristics as independent variables. Variables were selected based on theoretical considerations and forced into the model. *p* values and hazard ratios were indicated, knowing that the hazard ratios of covariates are interpretable as multiplicative effects on the hazard, and holding the other covariates constant.

## 5. Conclusions

This study demonstrates that AhR is expressed in the majority of BC tissues and may play differential roles in breast tumorigenesis according to both its subcellular location and the degree of metastatic progression. Indeed, only in LN-negative patients is the total AhR expression correlated with poor OS and appears as an independent prognostic factor. By contrast to nuclear AhR, cytoplasmic AhR has no prognosis value in our cohort. Moreover, the prognostic value of AhR might be reversed in LN-positive patients. Altogether, these data strongly suggest that AhR may be an interesting marker to identify high-risk BC patients, especially amongst those with early luminal BC and node-negative disease. Further validation studies also looking at the impact of adjuvant therapy on the observed prognostic impact are needed before definitive clinical conclusions can be drawn.

## Figures and Tables

**Table 1:** Patient clinicopathological characteristics.

Clinical and Pathological Characteristics ^a^	*n =* 302 ^b^	%
ER status		
Negative	58	19.2
Positive	244	80.8
PR status		
Negative	126	41.7
Positive	176	58.3
HER2 status		
Negative	265	87.7
Positive	35	11.6
Unknown	2	0.7
Triple negative		
No	264	87.4
Yes	36	11.9
Unknown	2	0.7
Histologic type ^c^		
Invasive lobular	40	13.2
Invasive medullar	11	3.6
Invasive mucinous	4	1.3
No Special Type (NST)	163	54
DCIS (only or with NST)	82	27.2
Unknown	2	0.7
Tumor size		
pT1	199	65.8
pT2	87	28.8
pT3	4	1.3
pT4	11	3.6
Unknown	1	0.3
Grade		
I	15	5
II	106	35.1
III	46	15.2
Unknown	135	44.7
Lymph node status		
Negative	162	53.6
Positive	124	41.1
Unknown	16	5.3
Local recurrence ^d^		
No	251	83.1
Yes	40	13.2
Unknown	11	3.6
Distant metastases ^d^		
No	232	76.8
Yes	59	19.5
Unknown	11	3.6

^a^ All tumor-related information refers to the primary tumor. ^b^ Five of 297 patients were bilateral primary BC. ^c^ NST includes the formerly called “Invasive ductal” and “other” types, and DCIS are Ductal Carcinoma In Situ ^d^ Local recurrence and distant metastases were detected during the follow-up.

**Table 2:** Distribution and correlation of total, nuclear, and cytoplasmic AhR expression in the whole cohort and in the lymph node-negative versus lymph node-positive BC.

	Whole Cohort (*n =* 302)	Lymph Node-Negative (*n =* 162)	Lymph Node-Positive (*n =* 124)
**Total AhR expression**			
Mean IRS +/− SE	6.66 +/− 4.85	6 +/− 4.56	6.85 +/− 4.30
Low expressing tumors *n* (%)	27 (8.9%)	12 (7.4%)	15 (12.1%)
High expressing tumors n (%)	275 (91.1%)	150 (92.6%)	109 (87.9%)
**Nuclear AhR expression**			
Mean IRS +/− SE	4.15 * +/− 2.92	4.10 * +/− 2.69	4.21 * +/− 2.52
Low expressing tumors n (%)	27 * (8.9%)	12 * (7.4%)	15 * (12.1%)
High expressing tumors n (%)	275 * (91.1%)	150 * (92.6%)	109* (87.9%)
**Cytoplasmic AhR expression**			
Mean IRS +/− SE	2.51 * +/− 2.67	2.41 * +/− 2.64	2.65 *+/− 2.28
Low expressing tumors n (%)	125 * (41.4%)	69 * (42.6%)	57 *(46%)
High expressing tumors n (%)	177 * (58.6%)	93 * (57.4%)	67 *(54%)
**Correlation between nuclear and cytoplasmic AhR**			
Correlation coefficient	0.539 ***	0.476 ***	0.618 ***
*p* values	5.66 10^−23^	1.57 10^−10^	2.09 10^−14^

The cut-off value between low and high expression was defined as an IRS ≥ 2 (total) or ≥ 1 (nuclear and cytoplasmic). Mean IRS and percentages of low and high expressing tumors were compared for nuclear and cytoplasmic expression in the whole cohort and in each sub-group. Differences or correlations were statistically significant for *p* < 0.05 (*) or *p* < 0.001 (***), using mean or percentage bilateral analysis and Spearman-Rho-Test.

**Table 3:** Correlations between AhR expression and clinicopathological characteristics and RIP140 expression, in the whole cohort and in the lymph node-negative versus lymph node-positive BC sub-groups.

	Whole Cohort (*n =* 167–302)	Lymph Node-Negative (*n =* 91–162)	Lymph Node-Positive (*n =* 71–124)
Age (diagnosis)	0.027	0.091	0.006
Histologic type	−0.061	−0.122	0.043
Tumor size	−0.055	−0.045	−0.059
Grade	0.055	0.029	0.052
Local recurrence	−0.020	−0.009	−0.028
Metastasis	−0.064	−0.086	−0.073
Triple negative	−0.045	−0.103	0.038
ER	0.024	0.047	−0.010
PR	0.071	0.078	0.061
HER2	0.010	0.079	−0.016
RIP140	0.284 ***	0.256 **	0.309 ***

Spearman Rho correlation coefficients are presented. Correlations were statistically significant for *p* < 0.01 (**) or *p* < 0.001 (***).

**Table 4:** Multivariate analysis of significant clinicopathological variables regarding OS in the whole cohort and in the lymph node-negative and lymph node-positive BC.

	*p* Value			Hazard Ratio		
	Whole Cohort	Lymph Node-Negative	Lymph Node-Positive	Whole Cohort	Lymph Node-Negative	Lymph Node-Positive
Age	0.000154 ***	0.004 **	0.002 **	1.033	1.041	1.039
Tumor size	0.000001 ***	0.061	0.005 **	1.479	1.345	1.348
ER	0.032 *	0.064	0.043 *	0.586	0.501	0.489
HER2	0.026 *	0.776	0.001 **	1.917	0.855	3.441
Total AhR	0.571	0.046*	0.454	1.164	3.369	0.788

Correlations were statistically significant for *p* < 0.05 (*), *p* < 0.01 (**), and *p* < 0.001 (***).

## Supplementary Materials

Supplementary materials can be found at https://www.mdpi.com/1422-0067/20/5/1016/s1. Figure S1: Patient overall survival according to AhR mRNA expression in lymph node negative versus lymph node positive BC.; Figure S2. Patient overall survival according to nuclear and cytoplasmic AhR expression in lymph node negative BC, according to RIP140 expression; Figure S3. Patient overall survival according to total AhR expression in lymph node positive BC, according to RIP140 expression. Table S1. Correlation between nuclear and cytoplasmic AhR and clinicopathological characteristics, nuclear receptors and coregulators in the whole cohort and in the lymph node negative *versus* positive breast cancers.

## Abbreviations

AhR	Aryl hydrocarbon receptor
ARNT	AhR nuclear transporter
BC	breast cancer
DFS	disease free survival
EMT	epithelial mesenchymal transition
ER	estrogen receptor
FISH	fluorescence in situ hybridization
HER2	human epidermal growth factor receptor 2
IRS	immunoreactive score
LCoR	ligand dependent corepressor
LMU	Ludwig Maximilians University
LN	lymph node
NCAD	N-Cadherin
NST	non-special type
OS	overall survival
PBS	phosphate buffered saline
pN	primary tumor node
PR	progesterone receptor
pT	primary tumor size
RAR	retinoic acid receptor
ROC curve	receiver operating characteristic curve
RIP140	receptor interacting protein of 140 kDa
RXR	retinoid X receptor
THR	thyroid hormone receptor
TNM status	tumor node metastasis status
TNBC	triple-negative breast cancer
VDR	vitamin D receptor
