# Supplementary material for: The Prognostic Impact of the Aryl Hydrocarbon Receptor (AhR) in Primary Breast Cancer Depends on the Lymph Node Status
Source: Int J Mol Sci. 2019 Feb 26;20(5):1016. doi: 10.3390/ijms20051016 (PMC6429124; doi:10.3390/ijms20051016)
Supplement: Supplementary file 1 [file ijms-20-01016-s001.pdf]

Appendix A

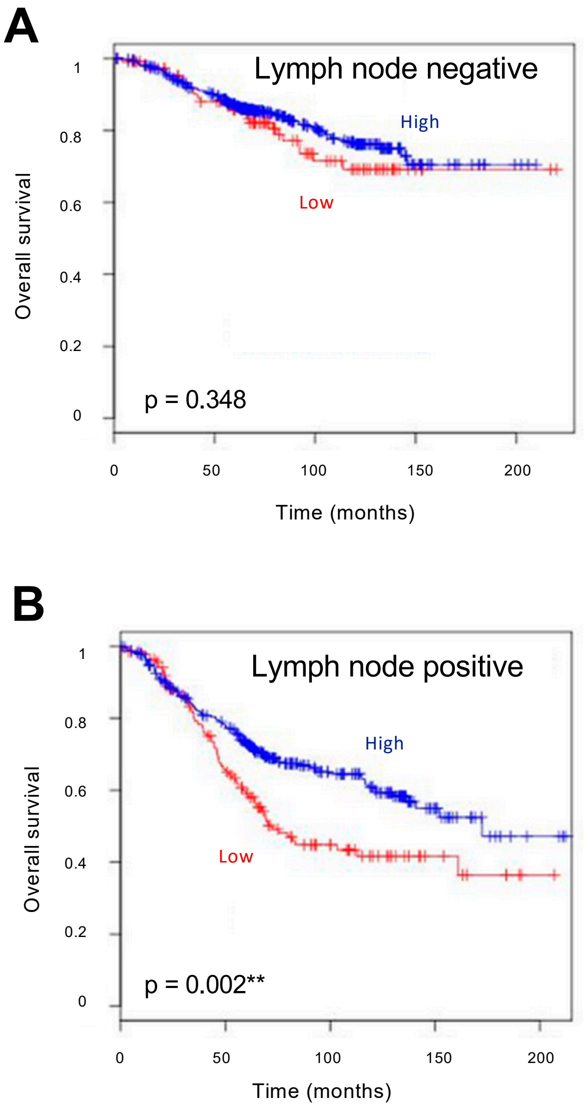

Supplementary Figure A1. Overall patient survival according to AhR mRNA expression in lymph node-negative versus lymph node-positive BC.

Kaplan–Meier analysis of the correlation between AhR mRNA expression with OS in lymph node-negative (A; *n* = 545) or -positive (B; *n* = 473) patients from a large dataset of breast cancer patients. The number of cases is indicated in each panel for low (in red) or high (in blue) AhR mRNA expression. Correlations were statistically significant for $p<0.01$ (**).

Appendix B

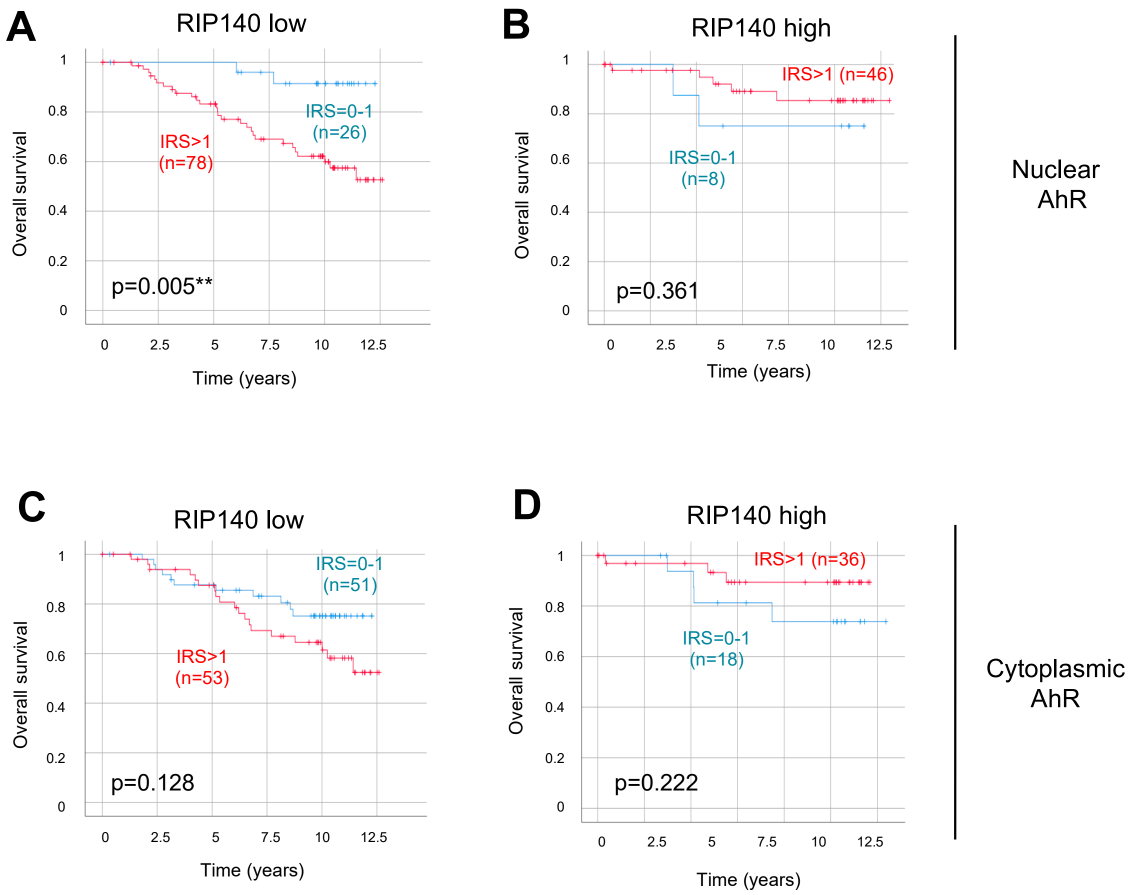

Supplementary Figure A2. Overall patient survival according to nuclear and cytoplasmic AhR expression in lymph node-negative BC, according to RIP140 expression. Kaplan–Meier analysis of the correlation between OS and nuclear (A and B) or cytoplasmic (C and D) AhR expression in lymph node-negative patients, with either a low (A and C) or high (B and D) RIP140 expression. The IRS cut-off value is above 4 for RIP140 and for AhR, and IRS cut-off values together with the number of cases in each arm are indicated in each panel, for low (in blue) or high (in red) AhR expression. Correlations are statistically significant for $p<0.01$ (**).

Appendix C

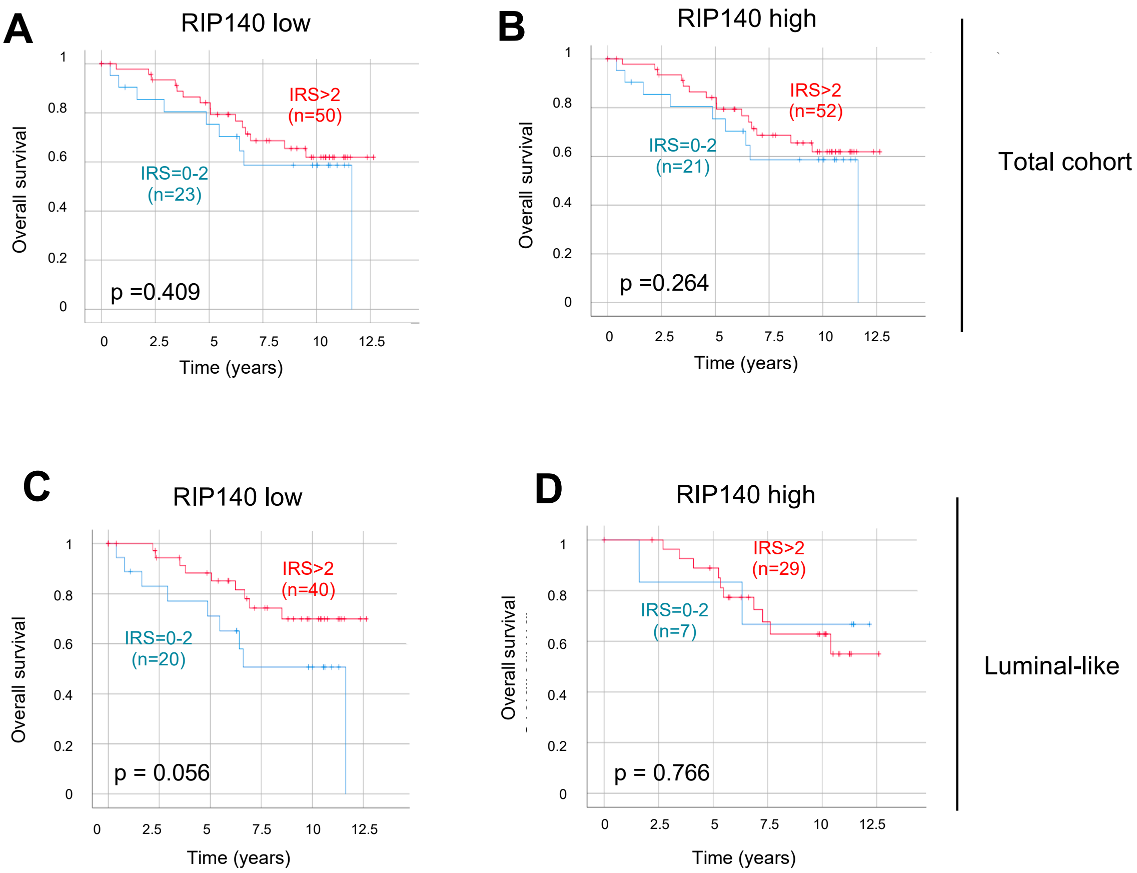

Supplementary Figure A3. Overall patient survival according to total AhR expression in lymph node-positive BC, according to RIP140 expression. Kaplan–Meier analysis of the correlation between OS and total AhR expression in lymph node-positive patients, with either a low (A and C) or high (B and D) RIP140 expression. The analysis has been performed with all lymph node-positive patients (A and B) and with only the luminal-like lymph node-positive patients (C and D). The IRS cut-off value is above 4 for RIP140 and for AhR, and IRS cut-off values together with the number of cases in each arm are indicated in each panel, for low (in blue) or high (in red) AhR expression.

Appendix D

Supplementary Table A1. Correlation between nuclear and cytoplasmic AhR and clinicopathological characteristics, nuclear receptors, and coregulators in the whole cohort and in the lymph node-negative versus -positive breast cancers

	Whole cohort (n = 167-302)		Lymph node-negative (n = 91-162)		Lymph node-positive (n = 71-124)	
AhR expression	Nuclear	Cytoplasmic	Nuclear	Cytoplasmic	Nuclear	Cytoplasmic
Age (diagnosis)	0.021	0.016	0.055	0.090	0.001	-0.006
Histologic type	-0.047	-0.051	-0.072	-0.121	0.038	0.050
pT (tumor size)	-0.100	0.002	-0.090	-0.010	-0.119	0.008
Grade	-0.007	0.111	-0.001	0.062	-0.029	0.123
Local recurrence	0.028	-0.072	0.068	-0.084	-0.016	-0.056
Metastasis	-0.095	-0.020	-0.061	-0.097	-0.140	0.012
Triple negative	-0.085	0.026	-0.125	-0.033	-0.042	0.137
ER	0.077	-0.055	0.067	-0.015	0.088	-0.120
PR	0.089	0.024	0.031	0.090	0.147	-0.057
HER2	0.076	0.043	0.072	0.065	-0.065	0.069
RIP140	0.239**	0.239**	0.260**	0.197*	0.250**	0.303**

Spearman Rho correlation coefficients are presented. Correlations were statistically significant for $p < 0.05$ (*) or $p < 0.01$ (**).
